# Mirroring Pain in the Brain: Emotional Expression versus Motor Imitation

**DOI:** 10.1371/journal.pone.0107526

**Published:** 2015-02-11

**Authors:** Lesley Budell, Miriam Kunz, Philip L. Jackson, Pierre Rainville

**Affiliations:** 1 Département de physiologie, Université de Montréal, Montréal, Québec, Canada; 2 Groupe de recherche sur le système nerveux central (GRSNC) and Centre de recherche de l’Institut universitaire de gériatrie de Montréal (CRIUGM), Montréal, Québec, Canada; 3 École de psychologie and CIRRIS and CRIUSMQ, Université Laval, Québec, Canada; 4 Département de stomatologie, Université de Montréal, Montréal, Québec, Canada; 5 Department of Psychology, University of Bamberg, Bamberg, Germany; University of Vienna, AUSTRIA

## Abstract

Perception of pain in others via facial expressions has been shown to involve brain areas responsive to self-pain, biological motion, as well as both performed and observed motor actions. Here, we investigated the involvement of these different regions during emotional and motor mirroring of pain expressions using a two-task paradigm, and including both observation and execution of the expressions. BOLD responses were measured as subjects watched video clips showing different intensities of pain expression and, after a variable delay, either expressed the amount of pain they perceived in the clips (pain task), or imitated the facial movements (movement task). In the pain task condition, pain coding involved overlapping activation across observation and execution in the anterior cingulate cortex, supplementary motor area, inferior frontal gyrus/anterior insula, and the inferior parietal lobule, and a pain-related increase (pain vs. neutral) in the anterior cingulate cortex/supplementary motor area, the right inferior frontal gyrus, and the postcentral gyrus. The ‘mirroring’ response was stronger in the inferior frontal gyrus and middle temporal gyrus/superior temporal sulcus during the pain task, and stronger in the inferior parietal lobule in the movement task. These results strongly suggest that while motor mirroring may contribute to the perception of pain expressions in others, interpreting these expressions in terms of pain content draws more heavily on networks involved in the perception of affective meaning.

## Introduction

How do we perceive the pain that others experience? There are many channels through which the emotional and sensory state of another person can be communicated to an observer. Vocalizations—such as “ouch!”, and gestures—such as a hand flinching away from a hot stove, are cues which can indicate that someone has experienced a painful stimulus. Another important cue, particularly in situations where the painful stimulus is internal or occurs out of sight of the observer, is facial expression.

In an earlier fMRI study looking at brain response to video clips of facial expressions of pain [1], we found that observation of dynamic facial expressions of pain elicited activation in the anterior cingulate cortex (ACC) and the anterior insula (aINS), two areas associated with the processing of the affective aspects of the pain experience in the self [2], as well as with the perception of pain in others [3,4,5,6,7,8,9].

However, we also noted activation of the inferior parietal lobule (IPL) and the inferior frontal gyrus (IFG); two regions theorized to comprise, along with the superior temporal sulcus (STS), a “core circuit” of the putative ‘human mirror neuron system’ (MNS), thought to contribute to the internal representation of observed actions and related socially-relevant phenomena [10]. It has been further suggested that it is the interaction of this core system with other networks for motor, sensory, and affective functions that supports various processes of social cognition, such as imitation, action understanding, language, and even emotion recognition and empathy [11]. Previous work looking at this system in humans has demonstrated activation of the IFG and/or IPL during both observation and execution, and/or imitation, of actions such as grasping or reaching for objects with the hands [12,13,14,15,16], facial movements such as chewing or biting [12], and even facial expressions of emotion [15,17,18,19,20]. Our earlier findings suggest that this mirror-type activity may also be involved in the perception of pain in others, via an internal motor simulation of a facial expression [1].

One question that has arisen in regards to motor mirroring is which areas might code for goals, intentions, or meaning of actions. In the context of an action such as grasping an object, evidence suggests that both the IFG and the IPL may be sensitive not only to these types of goals, but also the intention toward them [21,22]. However, these are transitive actions; goal-directed movements that involve the manipulation of external objects. Facial expressions of emotion are intransitive—while they may be produced in response to an external object, they indicate an internal state of the responder. This raises a fundamental question about the role of MNS regions in the coding of what an expression indicates—i.e. its emotional *meaning*—in addition to coding simply its motor aspects.

In our earlier work [1], not only did we observe these areas of the MNS to be recruited in the perception of facial expressions of pain, but their relative involvement in the process depended on whether subjects were explicitly attending to and evaluating the amount of pain they observed, or if they were performing a control task involving the discrimination of facial movements. More specifically, when subjects focused on estimating the amount of pain expressed in the videos, the IFG demonstrated stronger activity, whereas activity in the IPL was stronger when subjects focused on the movement of the facial features. Thus, these results revealed a dissociation between frontal and parietal regions possibly involved in processing emotional content of the meaning of the expression as opposed to mirroring facial movements, respectively.

However, making the claim of mirroring activity requires an experimental protocol that involves both the observation and the execution of a particular action. To this end, we designed a new protocol that included both observation and execution of pain faces. Subjects viewed a series of 1-second facial expressions of pain, and performed one of two tasks: express, using their own face, the amount of pain they perceived in the video clip (pain task), or imitate the facial movements (movement task). Importantly, we included a variable time-interval between the observation and execution events (i.e. delayed execution) in order to better separate the two functions.

In addition to replicating the previous results, the first main objective of the current study was to investigate overlapping activation in the IFG and the IPL for both observation and execution of pain expressions, and whether this activation would be stronger in response to pain expressions, versus neutral expressions. Our second main objective was to investigate the role of the IFG and IPL in the extraction of the meaning of the pain expressions. To this aim, we manipulated task demands, predicting a stronger response in the IFG when subjects focused on the affective meaning of the pain expression (pain expression task condition), and a stronger response in the IPL when subjects focused on the facial movements (movement imitation task condition).

## Methods

### Subjects

Participants were 23 healthy volunteers (13 women; 18–33 years old). Subjects were informed as to the purpose and procedures of the study, and written consent was obtained prior to the experiment.

### Ethics statement

The study was approved by the research ethics committee of the Centre de recherche de l’Institut universitaire de gériatrie de Montréal (CRIUGM) in Montréal, Canada.

### Stimuli

The stimuli used in this study were one-second video clips of facial expressions of pain, previously chosen and validated for our earlier study [1], and taken from a larger collection of facial expression stimuli created and validated in our laboratory [23]. The stimuli set comprised 96 clips portraying four levels of pain: neutral (pain 0), mild (pain 1), moderate (pain 2), and strong (pain 3), as produced by 8 actors and actresses, with 3 versions of each pain level per actor/actress.

### Experimental procedure

Prior to entering the scanner, subjects were instructed how to perform the two experimental tasks, and completed a brief practice session with ten additional stimuli not used in the actual experiment. For the pain expression task (pain task, PT)—subjects were instructed to express the amount of pain shown in the clip, using their own face (“use your own face to express the amount of pain that you see”). This was intended to induce stronger activation of a mental representation of the affective meaning of the facial expression and the involvement of a self-referential framework during production of the corresponding expression. For the movement imitation task (movement task, MT), subjects were instructed to imitate the facial movements shown in the clip, using their own face (“use your own face to imitate the facial movements yourself”). This was intended to emphasize the visuo-motor mapping of the expression. Although both tasks possibly involve automatic visuo-motor mapping as well as the automatic activation of mental representation of the meaning of the expression, the contrast between these tasks was expected to reveal brain regions more closely related to one process or the other.

**Fig 1:**
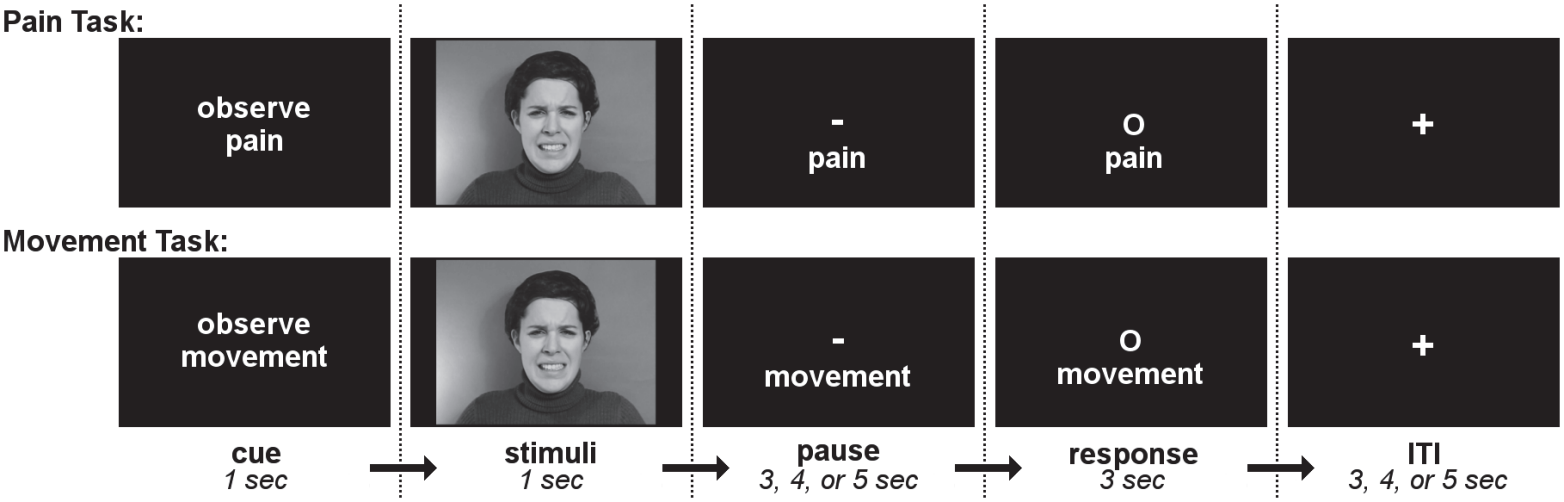
Trial structure for pain expression and movement imitation tasks. At the beginning of each trial, a one-second cue screen indicated which task—“pain” or “movement”—to perform on the upcoming clip. Next, the clip was presented (observation phase), immediately followed by a variable length pause of 3, 4, or 5 seconds, during which the screen displayed the cue word for the current task (“pain” or “movement”) below a dash symbol. This pause was followed by the response window of 3 seconds, during which the dash symbol was replaced by a circle, signaling the subject to begin their facial response (execution phase). Each trial ended with an ITI (inter-trial-interval) of variable duraction, during which subjects viewed a screen marked with a fixation cross.

Trial structure is shown in Fig. 1. At the beginning of each trial, a one-second cue screen indicated which task—“pain” or “movement”—to perform on the upcoming clip. Next, the clip was presented (observation phase), immediately followed by a variable length pause of 3, 4, or 5 seconds, during which the screen displayed the cue word for the current task (“pain” or “movement”) below a dash symbol. This pause was followed by the response window of 3 seconds, during which the dash symbol was replaced by a circle, signaling the subject to begin their facial response (execution phase). Each trial ended with an ITI (inter-trial-interval) of 3, 4, or 5 seconds, during which subjects viewed a screen marked with a fixation cross.

Each clip was shown twice, once per task, for a total of 192 trials, presented in 6 functional runs of 32 trials each. Within each run, the order of stimuli was pseudo-randomized according to pain level, actor gender and identity, and task, and the order in which the runs were presented was reversed for half of the subjects. The complete scanning session consisted of the 6 functional runs (8 minutes each) and an anatomical run (9:50 minutes).

After completing the imaging session, subjects exited the scanner and were brought into a separate room where they completed a rating session. The subjects were re-shown the clips using a laptop computer, and asked to rate each clip for the intensity of pain expressed, using a VAS from “no pain” to “worst pain imaginable.” To do this, subjects used the computer keyboard to position an on-screen slider bar on the VAS and to enter their response; the initial position of the slider at the start of each response window was randomized and the final position was recorded and linearly converted to a number between 0 and 100. The 92 clips were presented in a single block and their order within the block was randomized by the presentation software.

Stimuli presentation and the recording of subject responses during both the scanning and post-scanning test sessions were done using the E-Prime 1.2 presentation package (Psychology Software Tools, Inc.). An LCD Projector (EPSON, EMP-8300 XGA) was used to project the visual stimuli onto a screen inside the scanning chamber that subjects could view via a mirror positioned over the head coil.

### Behavioral responses: Facial Action Coding System

Subjects’ faces were videotaped during functional runs using an MR-compatible camera (MRC Systems, Heidelberg, Germany) mounted onto the headcoil. The camera captured the face of the subject as it was reflected by a mirror attached above the headcoil, and was positioned so as not to obstruct the visual field of the subject (as described in [24]). The onset of each response window was marked by an audio cue incorporated into the E-Prime presentation program that was recorded in the video but was not audible to the participant; this cue was used to identify the beginning of each 3-second response window for subsequent facial analysis.

Offline analysis of subjects’ facial displays during the response windows (execution phase) was done using the Facial Action Coding System (FACS; Ekman & Friesen 1987; see [25]), a finely-grained anatomically-based system that is considered the gold standard when decoding facial expressions and which involves the evaluation of the movement of different muscle groups—“Action Units” (AUs)—of the face. This analysis was used to verify that the responses matched the different pain intensities shown in the clips, to test for potential task differences, and to assess the similarity between the expressions produced by the subjects and those shown in the target clips. Facial movements produced in the 3-second response window were analyzed in each subject, and for both tasks, for a subset of two actors (one female, one male, randomly chosen) and including the four pain levels. FACS analysis was performed by two coders who were blind to the experimental conditions and trained by a certified FACS coder, using a software program designed for the analysis of observational data (Observer Video-Pro 9; Noldus Information Technology, NL). For the next stage of analysis, we selected the Action Units (AUs) that occurred in at least 5% of the coded segments and were more frequent during pain expressions, vs. neutral; this method is consistent with that used in previous studies (e.g. [26,27]). The frequency and intensity values of the selected AUs (AUs 4, 6, 7, 9, 10, 16, 25, 26, 43) were then combined into mean composite scores of pain-relevant facial responses [27]. The effects of task, and the pain intensity represented in the target clips on facial responses, were tested by entering these composite scores into a within-subject analysis of variance involving the factors type of task (pain vs. movement) and pain intensity (level 1–4). Furthermore, we assessed the similarity between the facial display produced by the subject and the expression shown in the target clips. This accuracy index was calculated by dividing the number of AUs that were shown by both the subject and the actor by the number of AUs that were shown in total, by both. Accuracy values were calculated separately for each pain intensity level and for each task, and were analyzed using analysis of variance (with two within-subject factors: type of task and pain intensity). This index was intended to verify that subjects produced more similar expressions in the movement imitation task than the pain expression task, consistent with the instructions of the movement task, which emphasized accuracy of imitation, vs. the instructions of the pain task, which emphasized transposition of the meaning of the expression onto a self-referential framework.

### Magnetic Resonance Imaging (MRI) equipment, data acquisition and analysis

Imaging was performed on a 3.0 Tesla whole-body scanner (Siemens TRIO), using an 8-channel headcoil at the Centre de recherche de l’Institut universitaire de gériatrie de Montréal (CRIUGM) in Montréal, QC, Canada. Blood oxygenation level-dependent (BOLD) signal was acquired using a standard T2*-weighted gradient-echo EPI sequence (TR = 3 sec; TE = 30 msec; FOV = 220 mm; flip angle = 90°; 64 × 64 mosaic matrix; 160 volumes; 40 interleaved, ascending, axial slices per whole-brain volume at 3.4 mm thickness; in-plane resolution of 3.44 × 3.44 × 3.4 mm nearly isotropic voxels). Structural images were acquired using a high-resolution, T1-weighted MPRAGE sequence (TR = 2.3 ms; TE = 2.91 ms; flip angle = 9°; FOV = 256 mm; matrix = 256 × 256; 1 × 1 × 1.2 mm voxels; 160 slices per whole-brain volume).

Processing of imaging data began with online inspection after each run for poor contrast, field inhomogeneity, major artifacts, or subject movement great enough to compromise the effectiveness of online motion correction. All subsequent data preprocessing and analysis was done using BrainVoyager QX (Version 2.2.1; Brain Innovation; Maastricht, Netherlands). Offline preprocessing of functional images included slice-time correction, motion correction and realignment, co-registration of each subject’s functional and anatomical volumes, spatial normalization (Talairach), spatial smoothing (8 mm FWHM Gaussian kernel), and high-pass temporal smoothing.

Statistical analysis of imaging data was performed using a general linear model (GLM) based on a canonical haemodynamic response function (HRF), which was used to model the expected BOLD signal change for each visual stimulus event and task response window. A GLM was created for each individual run/subject that included the following predictors: stimuli events defined by pain level (pain 0, 1, 2, and 3) and task (PT and MT); and response windows, also defined by pain level and task (i.e. 8 regressors of interest). Single-subject GLMs were then combined in group-level random-effect analyses.

In total, nine main analysis models are reported (Table 1). As the current study was designed to expand on an earlier work, the first four models were taken directly from our earlier study, to confirm the reliability of our previous findings in a separate group of subjects using the same stimuli and a similar methodology. To this end, the first two contrasts (Pain:Obs(PT) and Pain:Obs(MT)) were used to identify cortical areas responsive to observation of pain expressions (stimuli events); these involved a weighted contrast between all observed pain expressions and all observed neutral expressions in the PT (Pain:Obs(PT)), as well as the same subtraction contrast for the MT (Pain:Obs(MT)). Further, task differences in the stimulus-related responses were obtained by contrasting brain responses to all stimuli in one task versus the other (PT—MT (Obs) and MT—PT (Obs)).

**Table 1:** Contrast models used in the analysis of imaging data in this study.

Model #	Contrast name	Contrast description	Formula
1	Pain:Obs(PT)	Effect of pain expression in pain task, (PT), during stimuli event.	clip: pain(1,2,3)_PT_—pain0 _PT_
2	Pain:Obs(MT)	Effect of pain expression in movement task (MT), during stimuli event.	clip: pain(1,2,3)_MT_—pain0_MT_
3	PT—MT (Obs)	Task effect on stimulus-evoked responses: PT minus MT, during stimuli event.	clip: [pain(0,1,2,3)] _PT_—[pain(0,1,2,3)] _MT_
4	MT—PT (Obs)	Task effect on stimulus-evoked responses: MT minus PT, during stimuli event.	clip: [pain(0,1,2,3)] _MT_—[pain(0,1,2,3)] _PT_
5	Obs∩Exec(PT)	Overlap for observation and execution of facial expressions in pain task (PT) (conjunction)	clip ∩ response: [pain(0,1,2,3)] _PT_
6	Obs∩Exec(Pain;PT)	Effect of pain expression (vs. neutral) in pain task, (PT), across both stimuli and response events (conjunction).	clip ∩ response: pain(1,2,3)_PT_—pain0 _PT_
7	Obs∩Exec(Pain;MT)	Effect of pain expression (vs. neutral) in movement task (MT), across both stimuli and response events (conjunction).	clip ∩ response: pain(1,2,3)_MT_—pain0_MT_
8	PT—MT (Obs∩Exec)	Task effect on stimulus-evoked responses: PT minus MT, across both stimuli and response events (pain expressions only, no neutral) (conjunction)	clip ∩ response: [pain(1,2,3)] _PT_—[pain(1,2,3)] _MT_
9	MT—PT (Obs∩Exec)	Task effect on stimulus-evoked responses: MT minus PT, across both stimuli and response events (pain expressions only, no neutral) (conjunction)	clip ∩ response: [pain(1,2,3)] _MT_—[pain(1,2,3)] _PT_

Contrasts 1–4 replicate the analyses conducted in our previous study using a similar methodology [1]. Contrasts 5–9 test for overlap in brain activation across the observation and execution phases (conjunction) for all expressions in the pain task (5), for pain-related effects (pain vs. neutral) in the pain (6) and movement task (7), and for task effects (8: PT>MT; 9: MT>PT). Note: pain 0 = neutral; pain 1 = mild pain; pain 2 = moderate pain; pain 3 = strong pain; PT—pain task; MT = movement task; Obs = observation (clip); Exec = execution (response).

Our main analyses involved conjunction analyses investigating the overlapping effects of observation and execution of pain faces in the different conditions (conjunction of random effects). To this end, a first model looked at the observation and execution of facial expressions in the pain task, across all pain intensity levels (Obs∩Exec(PT)). The second model examined pain-related responses (weighted contrast of pain vs. neutral expression) that were common to the observation and execution (i.e. conjunction of stimulus and response phases of the task) in the pain task (Obs∩Exec(Pain;PT)), and in the movement task (Obs∩Exec(Pain;MT)). The third, and final, set of models looked for task effects (pain vs. movement) found in both observation and execution (PT—MT (Obs∩Exec) and MT—PT (Obs∩Exec)).

Although this article focuses on the main effects of pain and task across the observation and execution phases, a supplementary analysis was also performed to examine the interaction of pain and task during observation (models 1 vs. 2 from Table 1). A supplementary conjunction analysis of observation and execution was also performed for this interaction (models 6 vs. 7). These additional results are reported in S4 Table.

For all contrasts, a directed search was first conducted on a set of a priori areas, based on our previous work using a very similar methodology (Budell et al., 2010), including the ACC, INS, mPFC, IFG, and IPL leading to a total estimated search volume 250 mm^3^. The statistical threshold was adjusted to p ≤ 0.05 (corrected) and t ≥ 4.01, based on random field theory [28]. Additional peaks found at p ≤ 0.005 (t ≥ 3.25) were reported to protect against Type II error in the case of a priori areas. This was followed by a global search over the rest of the brain, using p ≤ 0.001 (uncorrected) and t ≥ 4.7.

## Results

### Behavioral results

**Fig 2:**
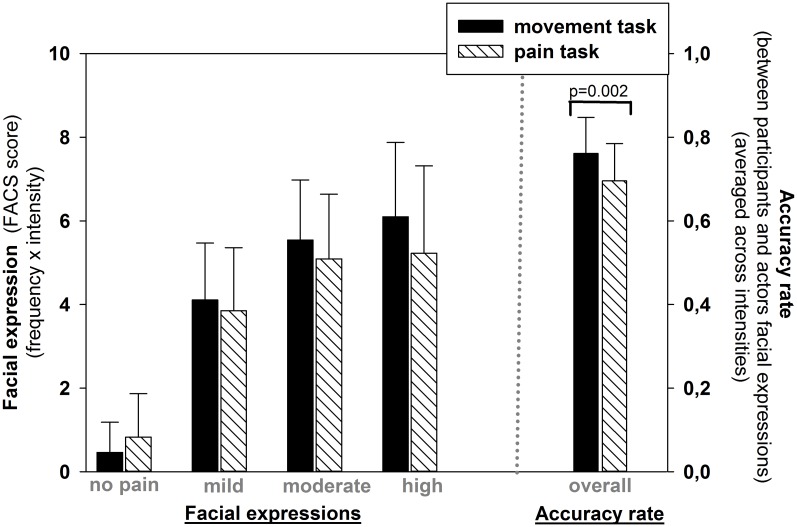
Results of FACS analysis of facial expressions. (A) Facial response, by intensity level, during subject responses. Results of FACS analysis of facial expressions shown by participants for different pain intensity levels, during response phase of both movement task and pain task conditions. ANOVA confirmed a main effect of pain levels (p < 0.001) but no significant effect of, or interaction with, task (p > 0.05). (B) Facial response accuracy. Facial responses displayed by the participants were more similar to those in the target expressions in the movement task condition, vs the pain task condition (p = 0.002).

Facial displays produced by participants, and assessed using the FACS, were compared across pain intensity levels and tasks. First, the overall amount of facial action produced during responses was comparable across the two task conditions (main effect of task: F(_1, 21_) = 1.33; p > 0.05). However, the amount of pain expressed increased significantly across pain levels (main effect of pain intensity: F(_3, 63_) = 120.02; p < 0.001). This effect of pain intensity was not significantly different between tasks (interaction: F(_3, 63_) = 2.62; p > 0.05), confirming that the participants’ responses adequately coded pain intensity in both tasks (Fig. 2). In addition, the accuracy index, which assessed the similarity between the expressions produced by the participants and those of the actors, confirmed that facial responses were more similar to the target expression in the movement imitation task condition than the pain coding task condition (MT—76.1% accuracy; PT—69.6% accuracy; main effect of task: F_(1,21)_ = 13.3; p = 0.002; see Fig. 2).

Analysis of the post-scan rating trials demonstrated that subject ratings for the amount of pain expressed (0–100; converted from VAS) matched the pre-defined levels: neutral/no pain (pain 0; mean ± SD = 15 ± 1.5), mild (pain 1; 23.5 ± 7.1), moderate (pain 2; 48.0 ± 9.2), and strong (pain 3; 72.1 ± 9.7). These results demonstrated that subjects not only differentiated significantly between the levels (F_(2, 35)_ = 838.5; p < 0.001), but they also perceived the intended pain level of the stimuli.

### Imaging Results

Observation of pain expressions: replication of effects of pain and task

Four initial contrasts were performed to confirm the reliability of the results from our previous study that formed the bases for the hypotheses of this study. The first two looked at observation of pain vs. neutral expressions in the pain task (pain-related response; Pain:Obs(PT)) and in the movement task (Pain:Obs(MT)), and the third and fourth contrasts were performed to compare activation during the observation of pain expressions in the pain task vs. movement task (PT—MT (Obs)and MT—PT (Obs)). Pain-related responses in the PT (Pain:Obs(PT)) confirmed the previous findings showing robust activation in ACC and aINS, which was not observed in the MT (Pain:Obs(MT)) (see S1 and S2 Tables). Task-related contrasts confirmed that the PT (PT—MT (Obs)) produced stronger activation in the midline medial frontal gyrus and the ACC, and in the left IFG, while the MT (MT—PT (Obs)) produced stronger activation bilaterally in the IPL (see S3 Table). The supplementary analysis of the interaction between pain and task, during observation, confirmed robust effects in several areas activated by pain expressions in the pain task (e.g. supracallosal ACC and IFG) and in the movement task (e.g. IPL) (see S4 Table).

Observation and execution of pain expression

**Fig 3:**
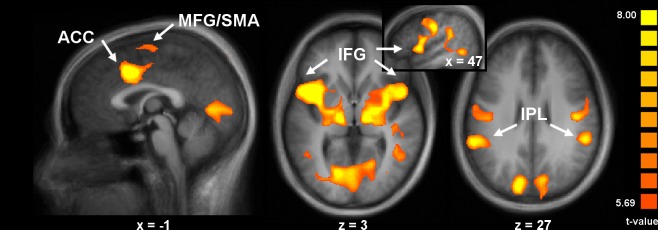
Areas commonly activated during observation and execution phases of the pain task (Obs∩Exec(PT)). Significant clusters are shown in the mACC, the SMA, IFG/aINS, and IPL (p < 0.001, uncorrected). Inset figure shows rostral-caudal extent of activation in the right IFG. See Table 2 for coordinates and peak t-values.

The first main objective of this study was to test if areas activated during the observation of pain expressions are also activated during the execution of pain expressions. A conjunction analysis was performed for the stimuli and response events, across all expression levels in the PT condition (contrast Obs∩Exec(PT); Table 2 and Fig. 3). Peaks of activation were found in a large midline cluster extending bilaterally, from the medial/superior frontal gyri to the supracallosal ACC (cingulate motor area; [29,30,31,32], bilaterally in the pre- and post-central gyri, and in large clusters including the posterior IFG (premotor, putative BA 44) and the aINS in both hemispheres. Additional peaks were noted bilaterally in the posterior part of the superior frontal gyrus (premotor), the precentral gyrus (PrCG; primary motor; putative face area), the right superior temporal gyrus (STG), and bilaterally in the IPL and intraparietal sulcus (IPS). Occipital areas where significant peaks appeared included the bilateral cuneus, occipito-temporal junction, and lingual gyrus. Subcortical activation included the bilateral thalamus, globus pallidus and putamen, and cerebellum. Although this was not a main goal of this study, the corresponding analysis performed on data acquired in the MT showed similar effects, indicating that this overlap between observation and execution was clearly not specific to the PT (not shown). However, there were very clear task differences in the magnitude of responses in several areas, as described below (see *Effects of task in observation and execution*).

**Table 2:** Effects of observation and execution of facial expressions in the pain expression task (including pain and neutral conditions).

Anatomical location	Hemisphere	BA	x	y	z	t-value
FRONTAL LOBE						
medial/superior frontal gyrus	R	6	5	-8	66	7.19
	R	6	5	1	63	6.69
anterior cingulate cortex (*posterior/supracallosal*)	MID	24	2	-3	42	6.72
	MID	24	-1	10	39	11.58
middle frontal gyrus	L	6	-49	4	42	7.32
precentral gyrus (*extending into postcentral*)	R	6/4	50	-11	36	8.83
	R	6/4	44	-17	33	8.97
	L	6/4	-43	-17	36	8.29
	L	6/4	-58	-5	15	8.28
inferior frontal gyrus (posterior)	R	44	47	7	3	9.17
	L	44	-49	10	6	11.14
INSULAR LOBE						
middle insula	R	13	29	1	9	9.94
	L	13	-37	1	6	10.08
anterior insula	R	13	47	7	3	9.17
PARIETAL LOBE						
inferior parietal lobule / intraparietal sulcus	R	40	32	-44	36	7.13
	L	40	-40	-50	36	7.43
	R	40	53	-38	24	8.77
	L	40	-58	-41	27	8.64
TEMPORAL LOBE						
middle temporal gyrus (posterior)	R	37	44	-53	0	7.81
	L	37	-43	-53	6	6.65
superior temporal gyrus (posterior)	R	22/42	50	-32	6	6.21
OCCIPITAL LOBE						
cuneus	R	19	8	-77	24	7.93
	L	19	-13	-80	30	9.06
lingual gyrus	R	17	17	-62	3	7.49
	MID/L	18	-4	-71	3	7.85
	L	18	-16	-71	3	8.62
SUBCORTICAL						
globus pallidus	R	–	17	-8	12	9.74
	L	–	-16	-5	15	8.97
putamen	R	–	20	-11	3	9.39
thalamus	R	–	11	-17	9	10.62
	L	–	-13	-17	9	10.41
cerebellum	R	–	32	-56	-21	10.13
	R	–	5	-71	-36	5.54
	L	–	-31	-59	-18	8.74
	L	–	-10	-68	-39	5.62

Peak values for areas of significant BOLD response change identified by conjunction analysis of stimuli presentation (clip) and task performance (response) (Obs∩Exec(PT)).

Note regarding identification and labeling of brain regions: coordinates for activation peaks are given in Talairach Space according to the Talairach atlas incorporated into the BrainVoyager QX software package. Brodmann area (BA) labels identified using the original Talairach atlas [64] and the online application for the Talairach Daemon (TD) database [65]. P ≤ 0.001 (uncorrected) unless otherwise indicated.

Observation and execution of pain versus neutral expressions

**Fig 4:**
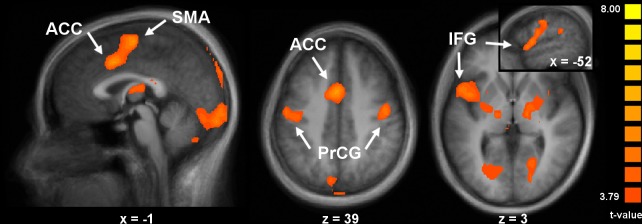
Effects of pain during both observation and execution of pain expressions (Obs∩Exec(Pain;PT)). A conjunction analysis of pain expressions, minus neutral expressions, during clip and response events, in the pain task condition revealed clusters in the ACC, SMA, the bilateral PrCG, and the left IFG/aINS (p < 0.001, uncorrected). Inset figure shows rostral-caudal extent of activation in the left IFG. See Table 3 for coordinates and t-values of peaks.

A complementary question related to our first objective was to investigate how pain content affected the mirroring response. To this end, we wanted to verify if pain-related responses (pain vs. neutral) during the observation of pain expressions are also activated during the execution of pain expression. A conjunction analysis was performed on stimuli and response events, after contrasting pain expressions minus neutral expressions in the PT (i.e. pain-related effect common to the observation and execution phases of the PT; contrast Obs∩Exec(Pain;PT); Table 3 and Fig. 4). Peaks of activation were found in a large midline cluster extending bilaterally from the superior frontal gyrus to the supracallosal ACC; bilaterally in pre- and post-central gyri, and in a cluster including the left posterior IFG (premotor, putative BA 44) and the aINS (similar, sub-threshold activation was also observed in the right IFG/aINS; see Table 3). Additional peaks were noted bilaterally in the supramarginal gyri of the IPL. Occipital areas where significant peaks appeared included the bilateral cuneus and lingual gyrus. Subcortical areas of activation included bilateral thalamus, globus pallidus and putamen, and cerebellum. The same conjunction analysis of pain-related responses across observation and execution phases of the MT (contrast Obs∩Exec(Pain;MT); Table 3) revealed significant effects only in the face area of the central region, as well as in the parietal operculum, lingual gyrus and thalamus.

**Table 3:** Effects of observation and execution of pain expressions (pain vs neutral).

Anatomical location	Hemisphere	BA	x	y	z	t-value
(A) PAIN EXPRESSION TASK						
FRONTAL LOBE						
medial/superior frontal gyrus	MID	6	-1	-11	60	5.08
anterior cingulate cortex (supracallosal)	MID	32	-4	7	39	5.85
precentral gyrus	R	6/4	44	-8	39	4.99
	L	6/4	-46	-17	36	5.13
	L	4/6	-49	-5	18	5.07
	L	4	-46	1	12	5.36
inferior frontal gyrus (posterior)	R	44	47	7	3	3.65*
	L	44/45	-49	4	9	5.03
	L	44/45	-52	10	3	4.90
INSULAR LOBE						
anterior insula	L	13	-40	13	6	4.77
middle insula	L	13	-43	4	3	5.21
PARIETAL LOBE						
inferior parietal lobule (supramarginal gyrus)	R	40	53	-32	24	4.18
	L	40	-55	-41	24	4.58
posterior cingulate cortex	MID	23	-1	-29	21	4.24
OCCIPITAL LOBE						
cuneus	L	19	-7	-77	36	4.50
superior occipital gyrus	R/MID	18/19	5	-95	27	5.24
lingual gyrus	R	19	17	-65	1	5.06
	L	19	-25	-71	6	4.65
SUBCORTICAL						
thalamus	R	–	17	-8	15	5.80
	L	–	-16	-8	12	5.75
	L	–	-13	-11	9	5.70
putamen	R	–	23	-2	12	5.95
	L	–	-25	-2	12	6.31
globus pallidus	R	–	17	-5	0	5.27
	L	–	-26	-8	0	4.80
cerebellum	R	–	11	-77	-18	6.10
	R	–	11	-92	-21	5.94
	R	–	32	-56	-24	6.83
	MID	–	2	-71	-39	4.21
(B) MOVEMENT IMITATION TASK						
FRONTAL LOBE						
precentral gyrus	R	6/4	35	-17	27	4.88
	L	6/4	-37	-14	30	4.67
PARIETAL LOBE						
inferior parietal lobule (supramarginal gyrus)	R	40	41	-32	27	4.03
postcentral gyrus	L	40	-43	-8	21	4.09
	L	40	-61	-20	15	4.25
OCCIPITAL LOBE						
lingual gyrus	R	17	17	-65	6	4.07
	L	17	-22	-74	6	4.49
SUBCORTICAL						
thalamus	R	–	14	-14	9	4.04
	L	–	-16	-17	9	4.45

Peak values for areas of significant BOLD response change identified by conjunction analysis of pain minus neutral during both stimuli presentation (clip/observation) and task performance (response/execution), in the (A) pain expression task condition (Obs∩Exec(Pain;PT)) and (B) movement imitation task condition (Obs∩Exec(Pain;MT)). See note in Table 2 regarding identification and labeling of brain regions.

*p < 0.002.

Effects of task in observation and execution

**Fig 5:**
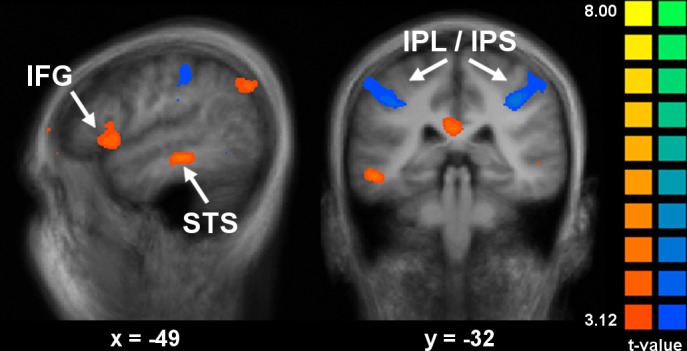
Effects of task during both observation and execution of pain expressions. For the pain task (PT—MT (Obs∩Exec); orange), a cluster of activation was observed in the left IFG, while bilateral clusters were observed in the IPL for the movement task (MT—PT (Obs∩Exec); blue) (p ≤ 0.005, uncorrected). Analysis included pain expressions only, no neutrals. See Table 4 for coordinates and t-values of peaks.

The most critical question of this study concerns coding of the meaning of the pain expressions, versus the facial movements contained in the expressions, during both the observation and execution of facial expression. We examined the differential activation induced by task (PT vs. MT) using a conjunction analysis across stimuli and response events (contrasts PT—MT (Obs∩Exec) and MT—PT (Obs∩Exec); Table 4 and Fig. 5). Positive peaks associated with the pain task (contrast PT—MT (Obs∩Exec)) were found in the midline medial/superior frontal gyrus, bilaterally in the IFG, and in the posterior cingulate cortex and precuneus. Note that the IFG peak, while located in putative BA 44, was slightly more anterior to the posterior IFG peaks found in the previous contrasts. Additional peaks were observed in the posterior portion of the middle temporal gyrus (MTG) and in the left superior occipital gyrus. Subcortical activation was observed bilaterally in the cerebellum. The conjunction of observation and execution in the reverse contrast (MT—PT (Obs∩Exec)) revealed activation in bilateral clusters extending along the fundus of the postcentral sulcus from the SPL into the IPL and IPS (Table 4 and Fig. 5).

**Table 4:** Main effects of task during both observation and execution (pain expressions only^†^):

Anatomical location	Hemisphere	BA	x	y	z	t-value
(A) PAIN TASK > MOVEMENT TASK (pain expressions)						
FRONTAL LOBE						
medial/superior frontal gyrus	MID	6	-4	10	60	4.98
inferior frontal gyrus	R	44	47	19	0	3.44* ^‡^
	L	44	-49	13	3	4.12*
TEMPORAL LOBE						
middle temporal gyrus (posterior portion)	L	21	-52	-32	-9	4.72
PARIETAL LOBE						
precuneus	L	31	-7	-74	30	3.93
posterior cingulate gyrus	L/MID	23/31	-4	-41	19	4.81
OCCIPITAL LOBE						
superior occipital gyrus	L	19	-43	-71	39	4.74
SUBCORTICAL						
cerebellum	R	–	35	-77	-42	4.29
	L	–	-31	-74	-42	4.57
(B) MOVEMENT TASK > PAIN TASK (pain expressions)						
PARIETAL LOBE						
inferior parietal lobule / intraparietal sulcus	R	40	35	-35	39	5.06
	L	40	-29	-41	39	4.88
	L	40	-55	-29	45	3.96*
postcentral gyrus	R	1/2/3	50	-23	36	4.70
	L	1/2/3	-58	-23	33	3.94*

Peak values for areas of significant BOLD response change during both the viewing and performance of pain expressions, in the pain expression task condition (A) (PT—MT (Obs∩Exec)), versus the movement imitation task condition (B) (MT—PT (Obs∩Exec)). See note in Table 2 regarding identification and labeling of brain regions.

*p < 0.002.

† Results are reported for pain expressions only. Similar results were obtained when including the neutral condition with only one exception, in the right IFG

‡: t = 2.31; not significant.

The supplementary analysis of the interaction of pain expression (vs neutral) and task showed effects in several regions during the observation phase, and some of these regions also showed a similar effect during the execution phase, as revealed by the conjunction analysis of observation and execution on the interaction term (see S4 Table). Areas showing stronger activation for pain expression (vs neutral) in the pain task included the left medial frontal gyrus, bilateral IFG, posterior cingulate cortex, left middle and inferior temporal gyri and the left cerebellum; whereas peaks of stronger activation for pain expression (vs neutral) in the movement task were observed bilaterally in the IPL/IPS. Peaks in the IFG and IPL/IPS closely matched those found in the conjunction of task effects.

## Discussion

The results of the current study reinforce, and move beyond, those of our earlier work in several important ways. For the first time, we investigated the overlap in brain response to the observation *and* execution of pain expressions, in an experimental protocol using dynamic pain expressions depicting different levels of pain. Importantly, the execution phase was separated temporally from the observation phase, allowing us to test the overlap (conjunction) between activation patterns with no overt motor confound in the observation phase and no overt visual confound in the execution phase. Results showed brain responses common to observed and executed facial expressions, consistent with “mirroring” properties and with previous studies on facial imitation [15,17,18,20,33]. More significantly, we investigated whether overlapping responses in different brain areas to both observation and execution of pain expressions may reflect either the processing of surface details of the expression, such as the movement or configuration of the facial features (movement task), or a deeper processing of the overall emotional meaning of the expression (pain task). This task contrast demonstrates that the decoding (observation) and the encoding (execution) of the meaning of the expression relies more strongly on the STS/MTG and the IFG, brain areas previously identified as responsive to biological motion [34,35,36,37,38] and motor observation and imitation [12,39], respectively, whereas attention to movement relies more on the IPL, another area previously identified as involved in the observation of motor actions [39,40]. Together, these findings support the broader notion that mirroring processes support perception of emotional expressions—pain expressions, in this case; and demonstrate how the neural representation of emotional meaning may be partly segregated from the representation of the facial movements that constitute the expression.

As in previous studies [3,5,6,7,8,9,41], including our own [1], we found that regions of the supracallosal ACC and bilateral aINS are activated in response to pain in others (Pain:Obs(PT); see S1 Table), but these effects were seen only when subjects were attending to the meaning of the expressions, and not when they attended to the motor aspects (Pain:Obs(MT); see S2 Table). The locations of the peaks we observed in these regions are consistent with the activation observed in response to acute self-pain [42]. Furthermore, we were able to replicate the functional dissociation between the IFG and IPL during the observation of pain faces [1], with the IFG being more robustly activated when subjects focused on the meaning of the pain expression, and the IPL being more engaged when subjects focused on the facial movements.

### Decoding pain expression: the role of mirroring

The first main objective of this study was to test whether areas of activation in the IFG and the IPL found during the observation of pain faces [1] were also involved in the delayed execution of pain expressions. Both regions are part of a putative human mirror neuron system [10], responding to both observed and executed actions. Although some studies have looked at the involvement of mirroring areas in the perception of facial expressions of emotions such as happiness, fear, disgust, sadness, or anger [15,17,18,20] and/or non-emotional mouth movements such as biting, chewing, or blowing out the cheeks [12,17], in most cases the execution phases of the experimental protocols occurred at the same time as the observation (i.e. imitation of the stimuli occurred simultaneously to the presentation of the stimuli) [15,18]; in only one study were the observation and execution phases temporally separated [17]. Here, we found robust, bilateral activation in the posterior IFG and the IPL, and we demonstrated significant involvement across observation and execution phases that were separated temporally in order to reduce confounding effects. This conjunction of stimuli and responses in the PT revealed not only the IFG and IPL, but also the ACC and aINS, areas which have been associated with affective mirroring for pain and disgust [1,3,6,7,43]. These results confirm our hypothesis that the IFG and IPL, two components of the ‘fronto-parietal human mirror neuron system’ [10], show mirror-type responses for pain expressions, and fit with the hypothesis that an IFG/IPL ‘core circuit’ of imitation interacts with the INS to form a system for affective mirroring that supports social cognition [10], perhaps via an effective link between the IFG and the aINS [44]. That we can demonstrate a common response of these areas during both observation and execution of pain expressions supports the notion that mirroring of both emotional states and motor movements underlies the perception of pain in others via facial expression.

Additionally, we identified an area of the supracallosal ACC and adjacent supplementary motor area (SMA) that showed common activation for both observation and execution. This area was revealed in our previous study as coding for pain (vs. neutral) and showing modulatory effects of perceived pain intensity [1]. In the current study, this area also demonstrated a greater response to pain expressions, during both observation and execution. This subregion of the supracallosal ACC has been discussed in relation to the control of movement in response to aversive stimuli such as pain [45,46], including motor facilitation of withdrawal, and inhibition of approach [47,48], as well as pain expression (Kunz et al., 2011). Interestingly, recent work has demonstrated involvement of this region during voluntary motor responses in both painful and non-painful contexts, but not in painful contexts lacking the motor response [49]. Together with the SMA, an area supporting motor control of movements [29] and movement preparation [50,51], ACC involvement in the response to signals of pain in others may reflect motor readiness or priming. Whether this priming is for withdrawal behaviors or pain expression [52,53], its occurrence in response to both observed and executed pain expressions is consistent with the concept of a mirroring mechanism.

### Extracting meaning from faces: task effects

The second main objective of this study was to investigate how the brain processes the meaning of a pain expression and the mirroring activations that occur during the observation of pain faces, by using two different tasks to direct attention toward either the pain communicated via the expression (i.e. meaning), or the constituent movements. Although each task required a different response from the subject—“express pain” vs. “imitate movements”—the stimuli in both conditions were the same, thus differences in the brain activation between tasks reflect the different processing requirements. As hypothesized, mirroring activity in the IFG and the STS/MTG was stronger when attention was directed to the meaning of the expression (pain task), and mirroring activity in the IPL was stronger when attention was directed to the movements. However, the greater IFG activation seen during the pain task was significant only when the neutral expressions were excluded from the analysis (PT—MT (Obs∩Exec); Table 4). Neutral expressions, in containing no pain, have less ‘meaning’ to consider, and thus there would be less difference in the task requirements for those stimuli. This interpretation is further supported by the observation that the IFG shows a significantly increased response to pain expressions (vs. neutral) in the PT trials, but not during the MT trials (see S1 and S2 Tables, as well as the interaction analysis results shown in S4 Table). These results are consistent with those of our previous studies, in which the IFG responded more strongly when subjects rated pain than when they evaluated facial movement [1], as well as when subjects evaluated images of limbs in painful, vs. non-painful situations, and images of pain, vs. neutral, facial expressions [54]. The IFG has been reported in several studies using facial movement and expressions [15,17,18,33], and a recent study has also implicated this area in the processing of semantic meaning of both speech and communicative gestures, finding greater IFG response when subjects listened to meaningful speech vs. an unfamiliar language, and when they viewed meaningful vs. nonsense gestures [55]. Importantly, the slightly more anterior position of this peak, compared with those of the other contrasts, is consistent with the idea that the function of the IFG is organized such that the more anterior portion is relatively more involved with the processing of semantic meaning, while the posterior portion is more involved with motor mirroring [56]. These results, together with those previously described, support the idea that the IFG is recruited more strongly when the meaning of a stimulus is being considered, not only in the case of pain expressions, but also in the broader context of other emotional expressions.

While most studies investigating imitation or observation and execution of emotional facial expressions have found a robust response of the IFG, the IPL is less commonly described. However, the IPL, as well as the SPL, have been widely reported in work looking at action observation and imitation [39], and the results of one meta-analysis strongly support a major role of both regions in imitation [57]. Here, bilateral IPL regions demonstrated strong common activation during both observation and execution of expressions in PT, consistent with the earlier findings on imitation. However, the IPL responded most strongly when subjects attended to the motor aspects of the expressions—i.e. the movements of the facial features—rather than when they attended to the affective content of the expressions (see as well the results of the interaction analysis, in S4 Table). Thus, while it is obviously involved when subjects attend to the meaning of the expressions, it may not be responding primarily to the emotional meaning of the expressions. Interestingly, there is evidence that the IPL may be involved in pain coding during hand-object interactions, responding more strongly when subjects grasped painful vs. non-painful objects [1]. However, another study found pain-related IPL response for images involving hands and feet, but not for facial expressions of pain [2], suggesting that motor-related IPL responses may be reinforced by salient consequences of an action (e.g. pain). In the current study, the lack of IPL response in the pain task could be due to the lack of information about such consequences. These results, together with those previously described, support the idea that the motor mirroring functions of these parietal regions may support, but are not sufficient for, the understanding of emotional expressions in others.

We also noted greater activation in visual areas for trials featuring pain faces (vs. neutral), as well as for PT trials (vs. MT), showing that the visual cortex responded more strongly not only to emotional stimuli, but also when attention was directed towards the meaning of the stimuli. Both cases may reflect the effects of positive feedback from higher-order executive areas, and is consistent with other work showing stronger responses of basic sensory regions to emotional stimuli [58]. It is possible that a similar positive feedback loop may also contribute to the increased response of regions to the pain expressions, such as the STS/MTG and the ACC/SMA complex.

### Potential limitations

In our previous study focusing on the observation phase, we noted stronger recruitment of the medial prefrontal cortex (mPFC) during the evaluation of pain, in comparison to the evaluation of movement. Here, task-related effects in this region were restricted to the most anterior part of the mPFC (see S3 Table). This region is theorized to have a major role in social cognition via the process of mentalizing, i.e. thinking about what others are thinking, and typically described in the context of intentions, thoughts, and beliefs, rather than feeling states or emotional states [59,60,61]. The fact that it was not similarly recruited for both observation and execution of the expressions in the present study is in line with an interpretation of mentalizing as a separate, yet complementary, evaluative process from emotional mirroring [see [62]].

Additionally, the inclusion of the execution phase in the current paradigm, while a strength of the study, introduces the potential confound of motor preparation or covert motor responses. These processes might produce motor-related activity during the observation phase. However, while they might contribute to any observed overlap between observation and execution, they do not compromise the interpretation of task differences.

Finally, we have approached the facial expression of pain only as an indicator of emotional state, and the subjects’ brain responses only as the decoding of an emotional message. This experimental paradigm does not allow us to consider the impact of pain decoding on the observer (i.e. pain communication as a transaction; see [63]). The brain responses we observed may in fact reflect a type of priming response that prepares the observer to act, either defensively against the threat of self-pain, or solicitously in the face of another’s need for aid. Future work in this area would thus benefit from additional investigation of effects of observed pain on subsequent actions, in order to investigate the potential influence these mirror-type responses have on intentions and motivational states, and to shed further light on the social function of pain expressions, beyond the simple communication of an affective state.

## Conclusion

In summary, we first confirmed findings from earlier studies that imply a role for the ACC and aINS in the perception of pain in others via facial expression. In addition, these regions, along with the IFG and IPL, were involved in both observation and execution of pain expressions, implicating all of these areas as parts of a broadly construed mirroring system for pain expression. The IFG and IPL have been proposed as a fronto-parietal “core circuit” for imitation which, in various combinations with other regions, form the neural basis for different functions of social cognition such as imitation and imitative learning, as well as empathy and affective mirroring [10]. Importantly, we found that IFG and IPL involvement was dependent on task requirements, with a functional dissociation between these two regions in the mirroring of emotional vs. motor components of pain expressions. The IFG, together with the MTG and STS/STG, two areas implicated in the perception of biological motion, were more strongly recruited when subjects attended to the meaning of the expressions, whereas the IPL was more strongly recruited when subjects attended to the movements. Together, these findings provide evidence that areas involved in motor mirroring contribute to a brain network underlying the perception of pain in others. However, the perception of the affective meaning in these expressions requires a deeper level of processing and more robustly recruits higher-order association regions involved in the perception of biological motion and semantic meaning. These results add to the growing body of research that suggests overlapping neural representations underlie the processing of both self and other experiences, allowing us to understand the internal state of another individual via the recreation of some aspects of that internal state within ourselves.

## Supporting Information

S1 Table Effects of pain expression during observation in pain expression task. Peak values for areas of significant BOLD response change identified by analysis of pain minus neutral, during stimuli events, in the pain task trials (Pain:Obs(PT)). Note regarding identification and labeling of brain regions: coordinates given in Talairach Space according to the Talairach atlas incorporated into the BrainVoyager QX software package. Brodmann area (BA) labels identified using the Talairach atlas [64] and the online application for the Talairach Daemon (TD) database [65]. P < 0.001 (uncorrected) unless otherwise indicated.
(DOCX)

S2 Table Effects of pain expression during observation in movement imitation task. Peak values for areas of significant BOLD response change identified by analysis of pain minus neutral, during stimuli events, in the movement task trials (Pain:Obs(MT)). See note in S1 Table regarding identification and labeling of brain regions.
(DOCX)

S3 Table Main effects of task during observation. Peak values for areas of significant BOLD response change during viewing of the facial expression stimuli in (A) the pain expression task condition (PT—MT (Obs)) versus (B) the movement imitation task condition (MT—PT (Obs)). See note in S1 Table regarding identification and labeling of brain regions.
(DOCX)

S4 Table Interaction of pain and task. Peak values for areas of significant BOLD response change for the interaction of pain and task: [pain(1,2,3)-pain(0)]PT—[pain(1,2,3)-pain(0)]MT, during the observation of pain expressions (Obs). See note in S1 Table regarding identification and labeling of brain regions. *p < 0.002. † Significant peak t-values are reported for the conjunction of observation and execution (Obs∩Exec) in the same structures as the peaks, or within the corresponding cluster of activated voxels, identified in the pain x task interaction during observation only (Obs).
(DOCX)
