# Supplementary material for: Mirroring Pain in the Brain: Emotional Expression versus Motor Imitation
Source: PLoS One. 2015 Feb 11;10(2):e0107526. doi: 10.1371/journal.pone.0107526 (PMC4324963; doi:10.1371/journal.pone.0107526)
Supplement: S1 Table — Peak values for areas of significant BOLD response change identified by analysis of pain minus neutral, during stimuli events, in the pain task trials (Pain:Obs(PT)). Note regarding identification and labeling of brain regions: coordinates given in Talairach Space according to the Talairach atlas incorporated into the BrainVoyager QX software package. Brodmann area (BA) labels identified using the Talairach atlas [64] and the online application for the Talairach Daemon (TD) database [65]. P < 0.001 (uncorrected) unless otherwise indicated. (DOCX) [file pone.0107526.s001.docx]

**Table S1. Effects of pain expression during observation in pain expression task.**

Peak values for areas of significant BOLD response change identified by analysis of pain minus neutral, during stimuli events, in the pain task trials (Pain:Obs(PT)). Note regarding identification and labeling of brain regions: coordinates for activation peaks are given in Talairach Space according to the Talairach atlas incorporated into the BrainVoyager QX software package. Brodmann area (BA) labels identified using the original Talairach atlas [[1](#_ENREF_1)] and the online application for the Talairach Daemon (TD) database [[2](#_ENREF_2)]. P < 0.001 (uncorrected) unless otherwise indicated.

| **Anatomical location** | **Hemisphere** | **BA** | **x** | **y** | **z** | **t-value** |
| --- | --- | --- | --- | --- | --- | --- |
| FRONTAL LOBE |  |  |  |  |  |  |
| superior /medial frontal gyrus | R/MID | 6 | 2 | -2 | 51 | 6.89 |
|  | L | 6 | -7 | -5 | 59 | 7.26 |
| superior frontal gyrus | L | 6 | -10 | 1 | 54 | 7.87 |
| ACC (supracallosal) | R | 24 | 3 | 7 | 42 | 5.48 |
|  | L | 24 | -4 | 7 | 39 | 6.32 |
|  | L/MID | 23 | -7 | -14 | 33 | 5.49 |
| precentral gyrus | R | 6 | 44 | -8 | 39 | 4.99 |
|  | L | 6 | -49 | -11 | 36 | 5.43 |
|  | L | 6 | -43 | 1 | 30 | 5.47 |
|  | L | 6 | -52 | 4 | 21 | 5.78 |
|  | L | 6 | -46 | 1 | 12 | 5.36 |
|  | L | 6 | -20 | -11 | 49 | 4.96 |
| inferior frontal gyrus | L | 44 | -49 | 10 | 0 | 5.56 |
| INSULAR LOBE |  |  |  |  |  |  |
| anterior insula | R | 13 | 34 | 7 | -5 | 5.58 |
|  | R | 13 | 29 | 4 | -9 | 7.90 |
|  | L | 13 | -37 | 13 | 3 | 5.37 |
|  | L | 13 | -40 | 4 | 0 | 5.60 |
|  | L | 13 | -34 | 0 | -9 | 5.77 |
| PARIETAL LOBE |  |  |  |  |  |  |
| inferior parietal lobule | R | 40 | 32 | -41 | 33 | 5.27 |
|  | L | 40 | -34 | -55 | 36 | 4.63 |
|  | L | 40 | -43 | -38 | 30 | 6.28 |
|  | L | 39/40 | -49 | -41 | 24 | 6.02 |
| posterior cingulate cortex | R | 23 | 14 | -26 | 33 | 4.95 |
|  | L/MID | 23 | -7 | -35 | 24 | 5.53 |
| postcentral gyrus | R | 1/2/3 | 38 | -21 | 33 | 4.30 |
|  | L | 1/2/3 | -43 | -23 | 33 | 5.15 |
| precuneus/paracentral lobule | R | 7 | 17 | -74 | 33 | 4.77 |
|  | L/MID | 7 | -7 | -74 | 36 | 5.08 |
| precuneus | R | 31 | 23 | -68 | 24 | 4.87 |
| TEMPORAL LOBE |  |  |  |  |  |  |
| superior temporal gyrus | R | 22/41/42 | 53 | -35 | 18 | 4.99 |
|  | R | 22/41/42 | 35 | -32 | 12 | 5.41 |
|  | R | 22/41/42 | 35 | -23 | 0 | 5.81 |
|  | L | 22/41/42 | -58 | -38 | 15 | 6.10 |
|  | L | 22/41/42 | -34 | -35 | 12 | 5.70 |
| superior temporal gyrus (temporal pole) | R | 38 | 32 | 7 | -18 | 8.57 |
|  | L | 38 | -43 | 13 | -18 | 7.57 |
| OCCIPITAL LOBE |  |  |  |  |  |  |
| lingual gyrus | R | 17 | 17 | -68 | 9 | 5.17 |
|  | L | 17 | -22 | -74 | 6 | 4.82 |
| middle occipital gyrus | R | 19 | 50 | -68 | -5 | 7.39 |
|  | L | 19 | -55 | -71 | -3 | 8.41 |
| inferior occipital gyrus | R | 18/19 | 35 | -86 | -21 | 8.25 |
|  | L | 18/19 | -31 | -92 | -21 | 7.91 |
| SUBCORTICAL |  |  |  |  |  |  |
| thalamus | R | – | 14 | -13 | 9 | 6.43 |
|  | L | – | -10 | -14 | 6 | 6.56 |
| globus pallidus/putamen | R | – | 17 | -22 | 6 | 10.52 |
|  | L | – | -19 | -2 | 6 | 10.03 |
| cerebellum | R | – | 32 | -56 | -24 | 6.83 |

**REFERENCES**

1. Talairach J, Tournoux P (1988) Co-planar stereotaxic atlas of the human brain : 3-dimensional proportional system : an approach to cerebral imaging. Stuttgart; New York: G. Thieme: Thieme Medical Publishers.

2. Lancaster JL, Woldorff MG, Parsons LM, Liotti M, Freitas CS, et al. (2000) Automated Talairach atlas labels for functional brain mapping. Hum Brain Mapp 10: 120-131.
