# Supplementary material for: Mirroring Pain in the Brain: Emotional Expression versus Motor Imitation
Source: PLoS One. 2015 Feb 11;10(2):e0107526. doi: 10.1371/journal.pone.0107526 (PMC4324963; doi:10.1371/journal.pone.0107526)
Supplement: S2 Table — Peak values for areas of significant BOLD response change identified by analysis of pain minus neutral, during stimuli events, in the movement task trials (Pain:Obs(MT)). See note in S1 Table regarding identification and labeling of brain regions. (DOCX) [file pone.0107526.s002.docx]

**Table S2. Effects of pain expression during observation in movement imitation task.**

Peak values for areas of significant BOLD response change identified by analysis of pain minus neutral, during stimuli events, in the movement task trials (Pain:Obs(MT)). See note in Table S1 regarding identification and labeling of brain regions.

| **Anatomical location** | **Hemisphere** | **BA** | **x** | **y** | **z** | **t-value** |
| --- | --- | --- | --- | --- | --- | --- |
| FRONTAL LOBE |  |  |  |  |  |  |
| medial frontal gyrus | R | 4 | 14 | -20 | 54 | 6.41 |
|  | L | 4 | -13 | -14 | 51 | 5.48 |
| precentral gyrus (extending into central sulcus) | L | 3/4/6 | -43 | -11 | 30 | 5.24 |
| PARIETAL LOBE |  |  |  |  |  |  |
| postcentral gyrus | R | 1/2 | 38 | -20 | 27 | 6.04 |
|  | L | 1/2 | -49 | -20 | 33 | 5.15 |
| inferior parietal lobule (supramarginal gyrus) | R | 40 | 38 | -35 | 27 | 5.74 |
|  | L | 40 | -43 | -32 | 24 | 4.69 |
| TEMPORAL LOBE |  |  |  |  |  |  |
| superior temporal gyrus (posterior portion) | R | 42/22 | 53 | -32 | 21 | 4.27 |
|  | L | 40 | -51 | -38 | 18 | 5.02 |
|  | L | 40/42 | -61 | -35 | 15 | 5.74 |
| superior temporal gyrus (temporal pole) | R | 38 | 35 | 16 | -21 | 8.10 |
|  | L | 38 | -40 | 16 | -24 | 7.41 |
| inferior temporal gyrus (occipitotemporal junction) | R | 37/39 | 50 | -65 | 0 | 5.47 |
|  | L | 37/39 | -55 | -68 | 0 | 5.51 |
| OCCIPITAL LOBE |  |  |  |  |  |  |
| lingual gyrus | R | 17 | 20 | -62 | 6 | 4.39 |
|  | L | 17 | -22 | -74 | 9 | 4.49 |
| SUBCORTICAL |  |  |  |  |  |  |
| globus pallidus / putamen | R | – | 17 | -2 | 3 | 7.00 |
|  | L | – | -25 | -5 | 3 | 7.29 |
| thalamus | R | – | 14 | -17 | 6 | 4.98 |
|  | L | – | -10 | -14 | 3 | 5.15 |
