# Supplementary material for: Mirroring Pain in the Brain: Emotional Expression versus Motor Imitation
Source: PLoS One. 2015 Feb 11;10(2):e0107526. doi: 10.1371/journal.pone.0107526 (PMC4324963; doi:10.1371/journal.pone.0107526)
Supplement: S3 Table — Peak values for areas of significant BOLD response change during viewing of the facial expression stimuli in (A) the pain expression task condition (PT—MT (Obs)) versus (B) the movement imitation task condition (MT—PT (Obs)). See note in S1 Table regarding identification and labeling of brain regions. (DOCX) [file pone.0107526.s003.docx]

**Table S3. Main effects of task during observation.**

Peak values for areas of significant BOLD response change during viewing of the facial expression stimuli in (A) the pain expression task condition (PT – MT (Obs)) versus (B) the movement imitation task condition (MT – PT (Obs)). See note in Table S1 regarding identification and labeling of brain regions.

| **Anatomical location** | **Hemisphere** | **BA** | **x** | **y** | **z** | **t-value** |
| --- | --- | --- | --- | --- | --- | --- |
| **(A) PAIN TASK > MOVEMENT TASK** |  |  |  |  |  |  |
| FRONTAL LOBE |  |  |  |  |  |  |
| superior frontal gyrus (dorsal) | R | 6 | 11 | 10 | 57 | 5.45 |
|  | L | 6 | -10 | 7 | 60 | 6.70 |
| superior frontal gyrus (lateral) | R | 6/8 | 17 | 22 | 51 | 5.02 |
|  | L | 6/8 | -10 | 25 | 51 | 4.51 |
| middle frontal gyrus | R | 6 | 38 | 10 | 45 | 4.80 |
|  | L | 6 | -28 | 10 | 45 | 5.15 |
| anterior cingulate (supracallosal) | L/MID | 32 | -1 | 25 | 33 | 4.15 |
| inferior frontal gyrus | L | 44 | -46 | 13 | 33 | 4.57 |
|  | L | 44 | -49 | 16 | 27 | 4.82 |
| medial frontal gyrus | R/MID | 10 | 2 | 55 | 6 | 5.16 |
|  | L/MID | 10 | -4 | 49 | -3 | 5.22 |
| PARIETAL LOBE |  |  |  |  |  |  |
| inferior parietal lobule | L | 39 | -40 | -71 | 36 | 8.11 |
| precuneus | L/MID | 23/31 | -4 | -56 | 30 | 6.40 |
| posterior cingulate gyrus | R | 31 | 2 | -47 | 27 | 5.46 |
|  | L | 31 | -7 | -41 | 27 | 5.7 |
| TEMPORAL LOBE |  |  |  |  |  |  |
| temporo-parietal junction | R | 39 | 44 | -65 | 30 | 6.78 |
|  | L | 39 | -55 | -62 | 24 | 10.63 |
| superior temporal gyrus | R | 41 | 41 | -20 | 6 | 4.71 |
|  | L | 41 | -41 | -23 | 6 | 5.51 |
| superior/middle temporal gyrus | R | 21/38 | 41 | 10 | -21 | 4.61 |
|  | R | 21/38 | 44 | 19 | -24 | 5.31 |
|  | L | 21/38 | -43 | 22 | -25 | 7.23 |
| middle temporal gyrus | R | 21 | 53 | -35 | 0 | 4.21 |
|  | L | 21 | -58 | -32 | -6 | 8.36 |
|  | L | 21 | -49 | -11 | -18 | 6.26 |
|  | L | 21/38 | -46 | 4 | -21 | 7.70 |
|  | L | 21 | -52 | 4 | -27 | 7.49 |
| inferior temporal gyrus | R | 21 | 53 | -2 | -30 | 4.26 |
| parahippocampal gyrus | R | 35/36 | 25 | -20 | -18 | 3.89 |
|  | L | 35/36 | -25 | -29 | -18 | 4.77 |
| OCCIPITAL LOBE |  |  |  |  |  |  |
| lingual gyrus | R | 30 | 26 | -47 | -3 | 5.07 |
| cuneus | MID | 18 | 0 | -74 | 24 | 6.11 |
|  | L | 18 | -7 | -71 | 24 | 6.65 |
| inferior occipital gyrus | R | 18 | 35 | -89 | -12 | 7.15 |
|  | L | 18 | -31 | -92 | -15 | 6.21 |
| SUBCORTICAL |  |  |  |  |  |  |
| cerebellum | R | – | 29 | -77 | -42 | 7.56 |
|  | L | – | -28 | -74 | -39 | 5.58 |
| **(B) MOVEMENT TASK > PAIN TASK** |  |  |  |  |  |  |
| FRONTAL LOBE |  |  |  |  |  |  |
| precentral gyrus | R | 6 | 62 | -2 | 27 | 3.84 |
|  | L | 6 | -52 | -2 | 30 | 4.37 |
| PARIETAL LOBE |  |  |  |  |  |  |
| postcentral gyrus | R | 2 | 59 | -26 | 45 | 5.21 |
|  | R | 3 | 59 | -17 | 33 | 6.11 |
|  | L | 1/2/3 | -64 | -20 | 39 | 4.44 |
|  | L | 3 | -58 | -23 | 33 | 4.77 |
| inferior parietal lobule | R | 40  40  40 | 35 | -35 | 39 | 4.99 |
|  | L | 40 | -40 | -32 | 39 | 5.42 |
|  | L | 40 | -28 | -41 | 36 | 5.40 |
