# Supplementary material for: Mirroring Pain in the Brain: Emotional Expression versus Motor Imitation
Source: PLoS One. 2015 Feb 11;10(2):e0107526. doi: 10.1371/journal.pone.0107526 (PMC4324963; doi:10.1371/journal.pone.0107526)
Supplement: S4 Table — Peak values for areas of significant BOLD response change for the interaction of pain and task: [pain(1,2,3)-pain(0)]PT—[pain(1,2,3)-pain(0)]MT, during the observation of pain expressions (Obs). See note in S1 Table regarding identification and labeling of brain regions. *p < 0.002. † Significant peak t-values are reported for the conjunction of observation and execution (Obs∩Exec) in the same structures as the peaks, or within the corresponding cluster of activated voxels, identified in the pain x task interaction during observation only (Obs). (DOCX) [file pone.0107526.s004.docx]

**Table S4. Interaction of pain and task.**

Peak values for areas of significant BOLD response change for the interaction of pain and task: [pain(1,2,3)-pain(0)]PT – [pain(1,2,3)-pain(0)]MT, during the observation of pain expressions (Obs). See note in Table S1 regarding identification and labeling of brain regions. *p < 0.002. † Significant peak t-values are reported for the conjunction of observation and execution (Obs∩Exec) in the same structures as the peaks, or within the corresponding cluster of activated voxels, identified in the pain x task interaction during observation only (Obs).

|  | **observation (Obs)** | | | | | | **conjunction (Obs∩Exec)†** |
| --- | --- | --- | --- | --- | --- | --- | --- |
| **Anatomical location** | **Hemisphere** | **BA** | **x** | **y** | **z** | **t-value** | **t-value** |
| **(A) PAIN TASK > MOVEMENT TASK** |  |  |  |  |  |  |  |
| FRONTAL LOBE |  |  |  |  |  |  |  |
| superior /medial frontal gyrus | R | 666 | 8 | 10 | 57 | 8.04 |  |
|  | L | 6 | -4 | 10 | 51 | 5.66 |  |
|  | L | 6 | -10 | 7 | 60 | 9.66 | 4.91 |
| middle frontal gyrus | R | 666 | 38 | 10 | 45 | 4.73 |  |
|  | L | 6 | -28 | 10 | 51 | 6.70 |  |
|  | L | 6 | -43 | 13 | 39 | 5.74 |  |
| precentral gyrus | R | 6 | 44 | -2 | 42 | 4.55 |  |
| ACC (supracallosal) | MID/L | 24/32 | -4 | 17 | 38 | 4.84 |  |
| ACC (posterior) | MID/L | 23 | -4 | -17 | 33 | 5.92 |  |
| inferior frontal gyrus | R | 4444 | 50 | 10 | 0 | 4.41 | 4.08 |
|  | L | 44 | -49 | 13 | 3 | 5.23 | 5.05 |
| PARIETAL LOBE |  |  |  |  |  |  |  |
| inferior parietal lobule | R | 77 | 38 | -62 | 30 | 6.18 |  |
|  | L | 7 | -34 | -65 | 36 | 5.70 |  |
| precuneus | L | 31 | -7 | -62 | 36 | 5.43 |  |
| posterior cingulate gyrus | MID/L | 23/30 | -1 | -41 | 21 | 6.46 | 3.81 |
| angular gyrus | L | 39 | -58 | -59 | 18 | 6.52 |  |
| TEMPORAL LOBE |  |  |  |  |  |  |  |
| superior temporal gyrus (temporal pole) | L | 38 | 35 | 4 | -21 | 5.26 |  |
| middle temporal gyrus (posterior portion) | R | 21 | 41 | -32 | -6 | 4.78 |  |
|  | L | 21 | -55 | -35 | -6 | 6.14 | 3.82 |
|  | L | 21 | -49 | -30 | -6 | 5.66 |  |
|  | L | 21 | -55 | -17 | -9 | 5.40 |  |
| inferior temporal gyrus (posterior portion) | L | 20 | -64 | -47 | -15 | 5.75 | 5.44 |
| inferior temporal gyrus (temporal pole) | R | 38 | 41 | 22 | -24 | 5.02 |  |
|  | L | 38 | -46 | 19 | -27 | 5.93 |  |
|  | L | 38 | -52 | -5 | -24 | 5.04 |  |
| OCCIPITAL LOBE |  |  |  |  |  |  |  |
| cuneus | L | 17 | -1 | -92 | 15 | 4.83 |  |
| inferior occipital gyrus | R | 18 | 39 | -78 | -8 | 4.47 |  |
|  | R | 18/19 | 26 | -83 | -21 | 7.43 |  |
|  | L | 18/19 | -31 | -92 | -15 | 6.08 |  |
| SUBCORTICAL |  |  |  |  |  |  |  |
| cerebellum | R | – | 32 | -65 | -24 | 5.06 |  |
|  | R | – | 29 | -53 | -30 | 5.07 | 4.12 |
|  | R | – | 26 | -74 | -36 | 4.66 | 3.66* |
| **(B) MOVEMENT TASK > PAIN TASK** |  |  |  |  |  |  |  |
| PARIETAL LOBE |  |  |  |  |  |  |  |
| postcentral gyrus | R | 1/2/3 | 59 | -14 | 26 | 4.28 |  |
| supramarginal gyrus | R | 40 | 59 | -17 | 33 | 5.83 |  |
|  | L | 40 | -55 | -23 | 30 | 3.51* | 3.52* |
| inferior parietal lobule / intraparietal sulcus | R | 7 | 35 | -35 | 42 | 4.81 | 4.49 |
|  | L | 7 | -46 | -29 | 42 | 4.77 | 4.33 |
